# Snap out stigma photovoice project in the U.S. South

**DOI:** 10.1186/s12913-022-08147-3

**Published:** 2022-06-20

**Authors:** Latrice C. Pichon, Andrea Williams Stubbs, Michelle Teti

**Affiliations:** 1 Division of Social and Behavioral Sciences, The University of Memphis, School of Public Health, 3825 Desoto Avenue | 209 Robison Hall, TN 38152 Memphis, USA; 2 Department of Public Health, The University of Missouri, 806 Lewis Hall, Columbia, MO 65211 USA

**Keywords:** Photovoice, Stigma, PLWH, Ending the HIV epidemic

## Abstract

**Background:**

The purpose of SNAP Out Stigma (SOS) was to design and implement a community-based intervention to reduce HIV-related stigma for people living with HIV (PLWH) in the Deep South. This region is a subset of 9 states including Memphis, Tennessee (project site) driving the epidemic in the United States. The purpose of this paper is to explain how PLWH in the U.S. South used Photovoice to communicate stigmatizing lived experiences and contextualize their intersection with multi-level sources of support.

**Methods:**

PLWH attended one-on-one and/or group sessions with other PLWH. In Session 1, PLWH received a project overview, met other participants, received a camera and camera training, completed a standardized internalized stigma scale, discussed experiences of internalized stigma, and were instructed to take 3–10 pictures that captured stigma. In Session 2, PLWH discussed the pictures and their meaning. In Session 3, PLWH expanded on what they shared in previous sessions in a one-on-one interview. Thematic analysis captured key patterns of how PLWH experienced stigma.

**Results:**

Forty-seven PLWH attended Session 1 and were issued a camera. Of those, 35 completed sessions 2 and 3. Participants self-identified as cis man who has sex with men (*n* = 18), ciswoman (*n* = 5), transwoman (*n* = 10), and non-binary (*n* = 2). Four emergent themes intersecting with internalizations of stigma were identified including: medical, social support, church, and self.

**Conclusions:**

The SOS intervention created a safe space for PLWH to share lived experiences of stigmatization. Photovoice facilitated discussion topics ranging from healing and recovery to overcoming factors of social determinants of HIV. We identified trauma-informed growth as an area of future programs for PLWH.

**Supplementary Information:**

The online version contains supplementary material available at 10.1186/s12913-022-08147-3.

## Background

Approximately 1.1 million persons in the United States are currently living with HIV and 42% of new infections in 2019 were among Black Americans [1]. Cisgender Black women and Cisgender Black men who have sex with men (MSM) experience the highest burden of new HIV diagnoses compared to other racial and ethnic groups [1]. Among people identifying as lesbian, gay, bisexual, transgender, queer (LGBTQ+) the disparities in HIV burden are most pronounced despite new scientific advancements in behavioral interventions and therapeutic treatments. Roughly 3.6 million LGBTQ+ adults live in the South [2]. In response to disparate incidence and prevalence in HIV, federal agencies across the U.S. Department of Health and Human Services launched Ending the HIV Epidemic: A Plan for America Initiative [3]. The goal is to reduce the number of new HIV infections by 2030. Shelby County, Tennessee was identified as 1 of the 48 counties with more than 50% of new diagnosis of HIV. Recent HIV surveillance reports show the overall prevalence is 7163 in Memphis metropolitan area (rate 530.3 per 100,000). The rate of diagnosis among Black adults and adolescents is 1277.7 per 100,000 compared to 205.8 per 100,000 among White adults and adolescents [4].

Despite the increased awareness of and access to HIV testing and treatment, HIV-related stigma continues to be a barrier to overall health and viral suppression. Medical systems of HIV care have known gaps in delivery of equitable care as patients report discriminatory treatment and experiences of stigmatization [5]. Disclosing HIV status to medical providers, friends, family, and sex partners may be met with shame, frustration, and limited social support [6]. Communities of faith have a history of perpetuating stigma particularly among LGBTQ+. Lack of support from multiple sources may often result in psychological trauma and mental health concerns.

Interventions designed to address stigma can create supportive environments to promote well-being among marginalized communities. A systematic literature review of 14 studies aimed to increase tolerance of people living with HIV (PLWH) and 3 studies that focused on improving coping strategies for PLWH found short term effects, but many gaps remain [7]. Particularly, interventions addressing internalizations of HIV stigma are needed. PLWH often internalize stigma by believing they are to be blamed, inducing shame, and increasing depression. Internalized stigma is significantly associated with poorer medication adherence and missed medical appointments. Another systematic review investigated intersections of intrapersonal, interpersonal, and community factors of HIV-related stigma and the impact on PLWH [8]. Study implications included the need for more culturally informed interventions addressing self-efficacy, self-esteem, and shame reduction.

One example of culturally tailored interventions includes church-based interventions which have found success increasing HIV knowledge and HIV testing, influencing individual-level HIV prevention behaviors, and reducing stigma [9–11]. The literature has identified lack of pastoral support, resource constraints, and HIV-related stigma as known barriers to implementing programming in faith communities [12]. However, fostering community engagement and increasing partnership development with Black religious congregations, Black clergy, local public health departments, community-based organizations, and academic institutions has the potential to circumvent known barriers and promote effective interventions [12, 13]. Such supportive spaces have significantly reduced HIV-related stigma, increased access to church-based testing and normalized HIV as a chronic health condition rather than a moral concern in predominantly Latino and Black churches [12].

Other potential places to reduce stigma include medical homes and HIV clinics with particular attention to clinic culture. Receipt of quality HIV care has significant challenges as Black MSM often experience medical distrust and have negative encounters with providers [14]. Offering cultural humility training to healthcare providers and toolkits on preferred language and pronoun usage may improve overall delivery of care. Cultural beliefs, misperceptions, and community norms around HIV testing and condom use are well documented. Peer norms for condom use and mental health wellness are related to Black MSM engaging in lower risk sexual behaviors [14]. Working with sero discordant couples and social support networks of PLWH to provide education on” knowing your HIV status” and promote messages of U=U may reduce stigma [15]. Finally, disentangling individual level psychosocial factors of resilience, denial, and coping mechanisms to improve mental health and overall well-being serves as a source of strength for PLWH [16]. Religion/spirituality and social support play an important role in HIV medication adherence. One study found praying once a day and satisfaction with social support received from individuals PLWH feel they can turn to in times of need were significantly associated with medication adherence [17]. Friedman and colleagues (2017) findings corroborate with the importance of social support and demonstrate the association with viral load suppression [18]. Higher levels of familial and other sources of support creates lower psychological distress among LGBTQ+ [19].

Creating an atmosphere of safety or ‘safe space’ can be facilitated by the adoption of photovoice interventions and methodologies in multiple settings. Photovoice is a participatory method developed by pioneering researchers, Wang & Burris [20], to engage marginalized communities to reflect upon lived experiences, participate in critical dialogue, and advocate for potential community change. This method has been used in multiple fields of study including public health, education, and social work to address community concerns such as youth violence prevention, physical disabilities, mental illness, and HIV [21–24]. Photovoice has the potential to promote critical dialogue about important issues like lived experiences through small and large group discussion of photographs taken by participants. The utility of photovoice is supported by increased individual empowerment, enhanced community engagement, and improved understanding of community need.

A study implemented in the Southern United States found photovoice as a facilitator for addressing individual- and community-level priorities affecting health behaviors and overall wellbeing among PLWH [25]. Research supports the use of photovoice to manage the mental and physical wellbeing of women living with HIV (WLWH) by providing an opportunity to process trauma in a creative and supportive environment [26]. Interpersonal-level group dynamics facilitated by photovoice processes have led to the ability for PLWH to express their fear, experience of stigma, and identity restoration while coping with an HIV diagnosis [27]. Reflecting on positive transformations through photovoice may also aid women in making and reframing perspectives of their experiences with HIV [28]. Through Picturing New Possibilities, a photovoice project among poor and racial/ethnic minority WLWH, participants experienced a process of empowerment that allowed them to practice control over life challenges [29]. In individual-level studies with cisgender women living with HIV in Philadelphia, photovoice facilitated HIV disclosure via strength and power to women revealing facial photos and selfies [29, 30]. Furthermore, evidence has shown photovoice can improve research practices and inform community level change and expanding networks for PLWH to engage local decision-makers, researchers, and media [24, 31].

While the extant literature is laying the foundation for the value and benefits of photovoice to participant growth and managing HIV, we have identified gaps in our understanding of the utility of these approaches to understanding stigma. HIV-related stigmas have multi-levels of influence but predominantly have been reported in homes and schools that ought to be supportive surroundings for youths and young adults aged 15 to 19 years old [32]. Further, normative sexual health behaviors among ethnic/racial minorities operate in a complex system of cultural influences, religious convictions, and family and community contexts [33]. The purpose of this paper is to explain how people living with HIV (PLWH) in the U.S. South used Photovoice to communicate stigmatizing lived experiences and contextualize their intersection with multi-level sources of support. Our longer-term goal is to educate communities and churches about stigma via their narratives and photo exhibitions.

## Methods

Memphis, Tennessee is among the largest cities in the Deep South and ranks 4th among all U.S. metropolitan statistical areas for new HIV infections. This city was identified in the U.S. Ending the HIV Epidemic (EHE) Initiative as a high HIV burden geographic area of focus. Interventions are needed to reduce HIV-related stigma via meaningful involvement of PLWH in decision-making processes. Applying principles of community-based participatory research approaches (CBPR), we worked closely with Headliners Memphis. This organization is a grassroots community advisory board of Black cis men who have sex with men (MSM), Black transwomen, and other allies living with or affected by HIV. We worked with Headliners to conceptualize SNAP Out Stigma - a project for PLWH to understand internalizations of stigma. We have worked intimately alongside The Headliners since 2015 and experienced firsthand accounts of the struggles and disparities encountered by this organization. We value this trusted partnership and strive to address social and structural issues affecting their community. Headliners often talk about a desire and longing to connect in spiritual settings but not being fully supported to do such because of their sexuality and HIV diagnosis. The project goal was to mobilize Headliners, implement a photovoice intervention for PLWH, and promote supportive environments for addressing stigma. We aimed to equip PLWH with skills to improve health outcomes and ultimately enrich their quality of life while also offering opportunities for Headliners to be involved in executive level decision-making for the design of the stigma reduction program. The University of Memphis Institutional Review Board (PRO-FY2019–363) determined this project non-human subject research. Data collection took place from August to December 2019 with written informed consent obtained from participants.

### Design, recruitment, sample

This project employed photovoice, a participatory method in which respondents identify, describe, and share their experiences with others via images [20] – particularly community stakeholders that can enact change based on participants’ needs. Given the goals of our project, we are focusing the photovoice intervention around participants discussing internalized stigma and intersections with supportive sources. Recruitment strategies consisted of approaching stakeholders at community meetings, one-on-one conversations, and community partner referrals to identify PLWH. We introduced the Snap Out Stigma photovoice project at weekly Headliners advisory board meetings. Flyers developed by a graphic designer from the LGBTQ+ community were shared with Headliners and posted on their Facebook page. The team distributed the flyer to community partner listservs (Connect to Protect Memphis C2P listserv and HIV Care and Prevention Group HCAP.) Both are distribution lists including HIV social and medical providers and PLWH. Finally, flyers were passed out at community events known to be frequented by individuals living with or affected by HIV. We recruited eligible participants for the photovoice intervention who self-disclosed as a person living with HIV (PLWH). Per feedback from our advisory board, we adjusted our previously more rigid eligibility criteria to be more inclusive of multigenerational, ethnic, sexual minority, and/or heterosexual groups representing the Memphis PLWH community. During the informed consent process, photovoice ethics was discussed due to the vulnerable nature of the participants living with HIV [see Data Collection Procedures]. The University of Memphis Institutional Review Board (PRO-FY2019–363) determined this project non-human subject research.

### Materials

Upon completion of the informed consent process, eligible participants completed a basic demographic self-administered questionnaire (e.g., age, race, sexuality), shortened HIV stigma scale [34], and items from the Duke Transformative Grantee survey provided by the funding agency. Then participants were issued a digital camera to keep, provided instructions on use, and practiced taking photos. Next, participants had a group discussion about the definitions of internalized stigma and were assigned homework to complete by the next group session. We used a semi-structured interview guide during group session 2 to facilitate the discussion on the images taken and abridged version of the ShOWED method [26]. During individual interviews we asked more in-depth questions summarized in Table 1. The discussion guide developed for this study is provided as Additional File 1.

**Table 1:** Sample interview guide for Snap Out Stigma project in Memphis, TN

Group Discussion	What do you **s**ee here?
	What is **h**appening?
	How does this relate to **o**ur lives?
	**W**hy does the problem or strength **e**xist?
	What can we **d**o about it?
Individual Discussion	How would you describe your experience participating in the project to someone else?
	Describe the experience of watching other people view your photos.
	What did you learn about the role of the church in your life and stigma?
	What could the church do to be more supportive in ending stigma?

### Data collection procedures

PLWH attended one-on-one and/or group sessions with other PLWH at a convenient time and location in the community (e.g., CBOs, home) between September 2019 and December 2019. In Session 1, PLWH completed the informed consent process, received an overview of the project integrating photovoice ethics [35], met other participants, received a camera and camera training, completed a standardized internalized stigma scale, discussed experiences of internalized stigma, and were instructed to take 3–10 pictures that capture images of stigma. The project team and the participants established ground rules including: no nudity, no pictures of participant actively engaging in illegal activity, and the importance of obtaining permission to take pictures of someone else’s face. We also helped participants to understand which pictures would reveal or protect their identity, based on their desire and intention. In Session 2, PLWH shared their pictures and discussed their meaning. We conducted seven focus group discussions of images ranging from 3 to 5 participants each with a final total sample size of *n* = 31 PLWH – which was sufficient to reach saturation or repeated ideas and themes [36], which occurred after focus group 6. Two participants were unavailable to attend due to scheduling conflicts and two participants had privacy concerns. In Session 3, PLWH (*n* = 34) clarified or expanded on what they shared in previous sessions in a one-on-one interview and made final selections for the planned display. Final photo displays with participant-chosen quotations were showcased in 4 church-based and 3 community-based photovoice exhibits centered around HIV National Observance Days and reaching over 400 congregation and 125 community members. All sessions lasted approximately 2 hours and were audio recorded and transcribed verbatim. Handwritten field notes were taken by the lead author and discussions were facilitated by the second author with expertise in Motivational Interviewing. A licensed clinician was also present in case PLWH experienced emotional distress.

### Investigative team credentials

The first author served as the Principal Investigator, who has lived in the Deep South for nearly 15 years. She oversaw the administration of the grant and execution of the photovoice project as well as taking field notes. The second author is a native Memphian employed in an HIV clinic for over 14 years and served as the liaison between patients living with HIV, medical providers and public health community and research partners. She has expertise in motivational interviewing and facilitated interviews and discussion groups. Both are Black cis gender Christian women. The third author is an expert in qualitative research and using photovoice methodology. She is a cis gender agnostic White woman from the Northeast. She has substantial experience implementing photovoice with sexual gender minorities, cis women, and PLWH. Her primary role was overseeing data analysis.

### Data analysis and coding

All discussion groups/interviews were audio-recorded, transcribed verbatim by a professional transcription company, and verified by the first author who simultaneously read each transcript and spot checked unclear areas by listening to select audio. Identifying information was removed from transcripts prior to data coding and analysis. Data were managed with Atlas.ti and analyzed using thematic analysis guided by the steps and principles developed by Guest [37] to capture the key patterns in the way that the participants talked about HIV-related internalized stigma, which was in line with our analysis goals. To conduct the analysis, first, each member of the coding team (*n* = 4) independently reviewed the transcripts to identify and discuss all possible patterns within and across data sources. Next, the team met and agreed upon an initial set of key themes based on the common patterns all reviewers found in the data. They defined these themes in a codebook. Then, they conducted initial coding. Coders met monthly to discuss and compare ideas, refine the codebook with additional definitions and example representative quotes as needed, and seek agreement or understanding on coding. Next the coders met to review their initial coding report, collapsing and expanding themes as appropriate based on the breadth and depth of initial themes and connections between the themes.

Finally, the group created a matrix report that identified each of the final themes with example quotes. The two facilitators (LP and AS) worked with each participant individually to select their top 3 photos. Together the analytic team triangulated the text from transcripts with photos to tell a cohesive story for each participant’s final display by adding photos to the matrix described above.

Trustworthiness of the data was assured by peer debriefing or discussions of the analysis, particularly around areas of disagreement, member checking or reviewing the data with participants as they chose and approved photos and text for analysis, and rich data and multiple quote and photo examples [36].

## Results

A total of 47 PLWH attended Session 1, completed the informed consent and stigma survey, and were issued a camera. Of those, 35 completed sessions 2 and 3 and included their final photo display in the exhibits. Most (66%) self-identified as Christian, and nearly 40% attended church or a place of worship weekly. PLWH worried about church rejection (34%), felt guilty (21%), and ashamed (34%). Participants self-identified as cis MSM (*n* = 18), ciswoman (*n* = 5), transwoman (*n* = 10), and non-binary (*n* = 2). Participant discussions of stigma uncovered how support was a complex issue in their lives and came at a cost. For example, participants identified four key areas in which support intertwined with stigma and complicated their use and access to external and internal resources. Four themes emerged: medical care, social support, church, and self. Each theme is described below in detail.

### Medical care as support and stigma

In general, PLWH in our cohort agreed HIV is no longer a death sentence as they recognized they are living proof of all possibilities. Participants self-disclosed the time frame of their diagnosis ranging from 2 years ago to at least 30 years. The importance of medication adherence facilitates improved health outcomes as expressed by a participant:

It’s not a death sentence. You can live as long as you want with HIV as long as you take your medicine every day. It’s been 2 years with me and you see I am moving on, staying positive. (transwoman).

Taking a daily pill was perceived as low burden as well as going to regular doctor appointments. Examples were shared by PLWH on how treatment gave them a sense of being in charge of their health and giving them a new outlook on life.

**Fig. 1:**
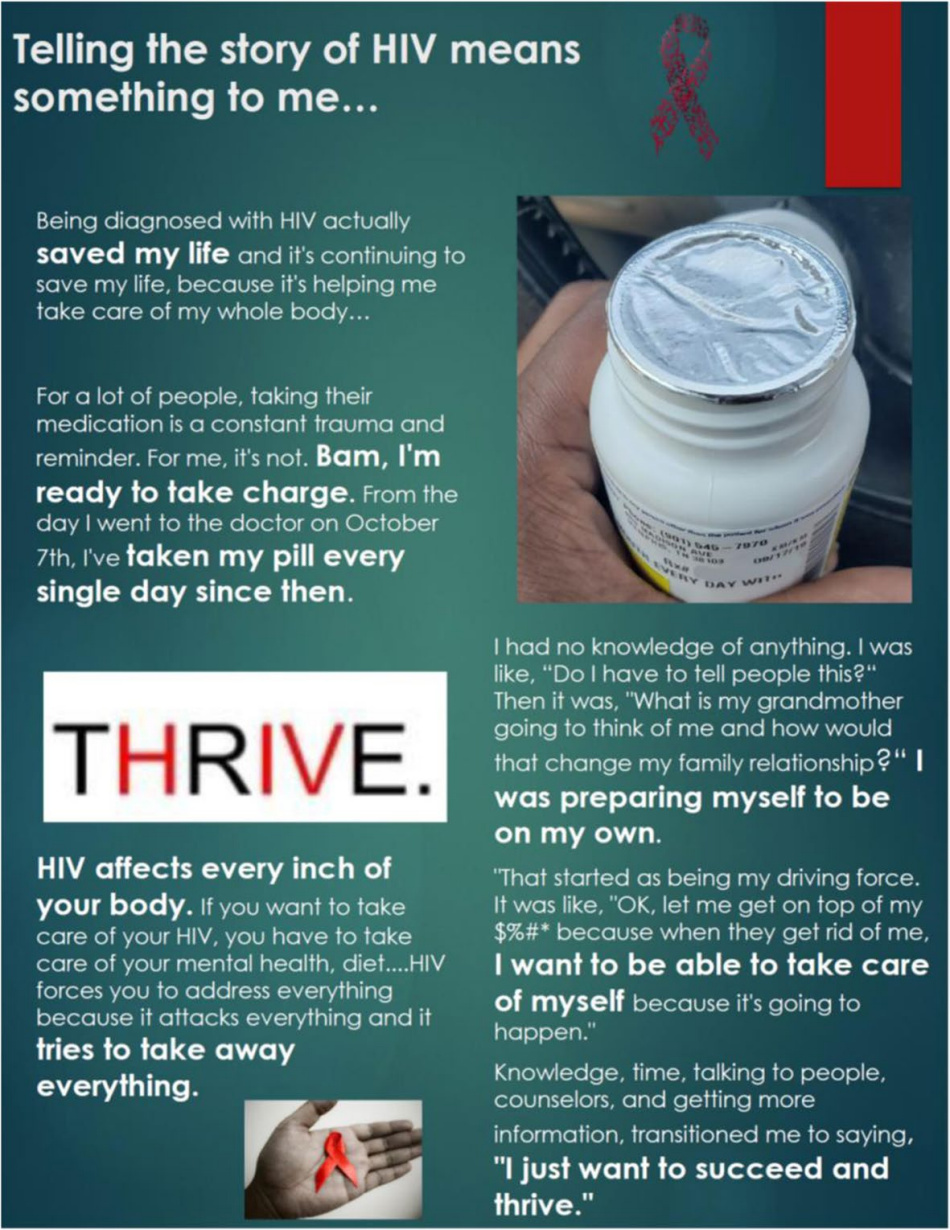
Telling the story of HIV means something to me

If I don’t take these two little pills every single day like clockwork for the rest of my life. I can’t play. I can’t work. I can’t love. I can’t get to that marriage or the kids. It’s my lifeline unfortunately. (cisgender male) (Fig. 1).

For others, the path to acceptance of an HIV diagnosis is not always as easy and in fact more complex. Taking daily medication can be a “constant trauma and reminder” of poor treatment received in medical settings and fear of being ‘outed’ as one participant explained:

Even though medicines can help you live a long time, sometimes because of how you’re treated, how you feel can make you even consider taking your own life or doing some other hard stuff to cope. (cisgender male).

There were descriptions of medical providers at the local health department interrogating participants while taking sexual history about time spent in jail, sex partners, and accusations of having sex with underage minors. One participant recollected their visit:

The lady was loud with the door cracked … I was thinking … ‘people don’t want to come back here once they hear someone talking to them like that.’ People will say, ‘I’ll stay away and just go and die.’ (cisgender male).

Lack of privacy and physical safe space further exacerbated feelings of shame of others seeing them at HIV care clinics for medical appointments or fear that others would find out they were living with HIV.

### Social support as support and stigma

PLWH discussed both family and peers as sources of support and in some cases facilitators of medication adherence and eventual undetectable viral load. Familial ties give power to deal with others who do not support PLWH and facilitate coping mechanisms. One participant said:

The thing that helped me become undetectable as soon as I did was sticking to my medication regimen … and I had friends that were [HIV] positive that were encouraging me. They were like, ‘It’s going to be OK. You’ll be fine.’ … It was having camaraderie and family around you saying, ‘You’ll be OK. Everything is going to be OK...’

With support from an interpersonal network, PLWH were able to disclose their status and work toward healthier behaviors to improve HIV outcomes.

In other scenarios, family and peers particularly from the LGBTQ+ community were less supportive. Internalizations of being shamed for life choices on who you love and blamed for HIV diagnosis emerged. A cisgender male told his story of dialogue with his mother:

People condemn you for the way you are living your life. They want you to change who you choose to love. My mother said: ‘You got the virus, and it’s probably because of the way that you are living.’ They just want to preach to you. It’s an everyday struggle. It’s not just today. I feel like I hear these words every day.

PLWH desire genuine love. There were protective mechanisms described on HIV disclosure to minimize the internalizations of hurt from loved ones as one participant shares:

[Telling people and having them reject me] started as being my driving force. It was like, OK, let me get on top of my $%#* because when they get rid of me. I want to be able to take care of myself because it’s going to happen.

**Fig. 2:**
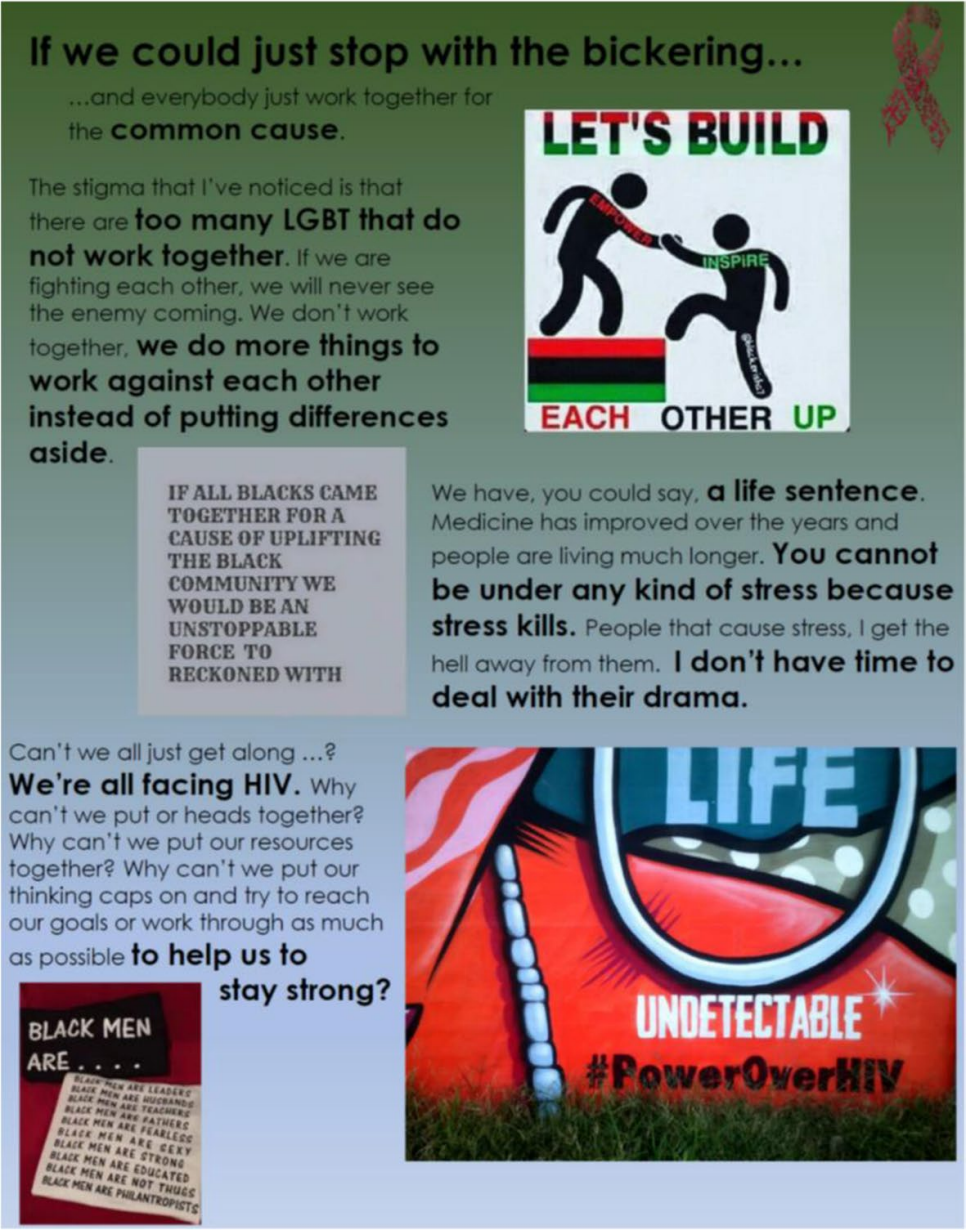
If we could just stop with the bickering

The LGBTQ+ peer network was described as both a source of strength and support as well as stigmatizing. Within network disagreements, presented barriers to working together as a cohesive community of LGBT to armor up to “see the enemy coming.” ( Fig 2).

### Church as support and stigma

**Fig. 3:**
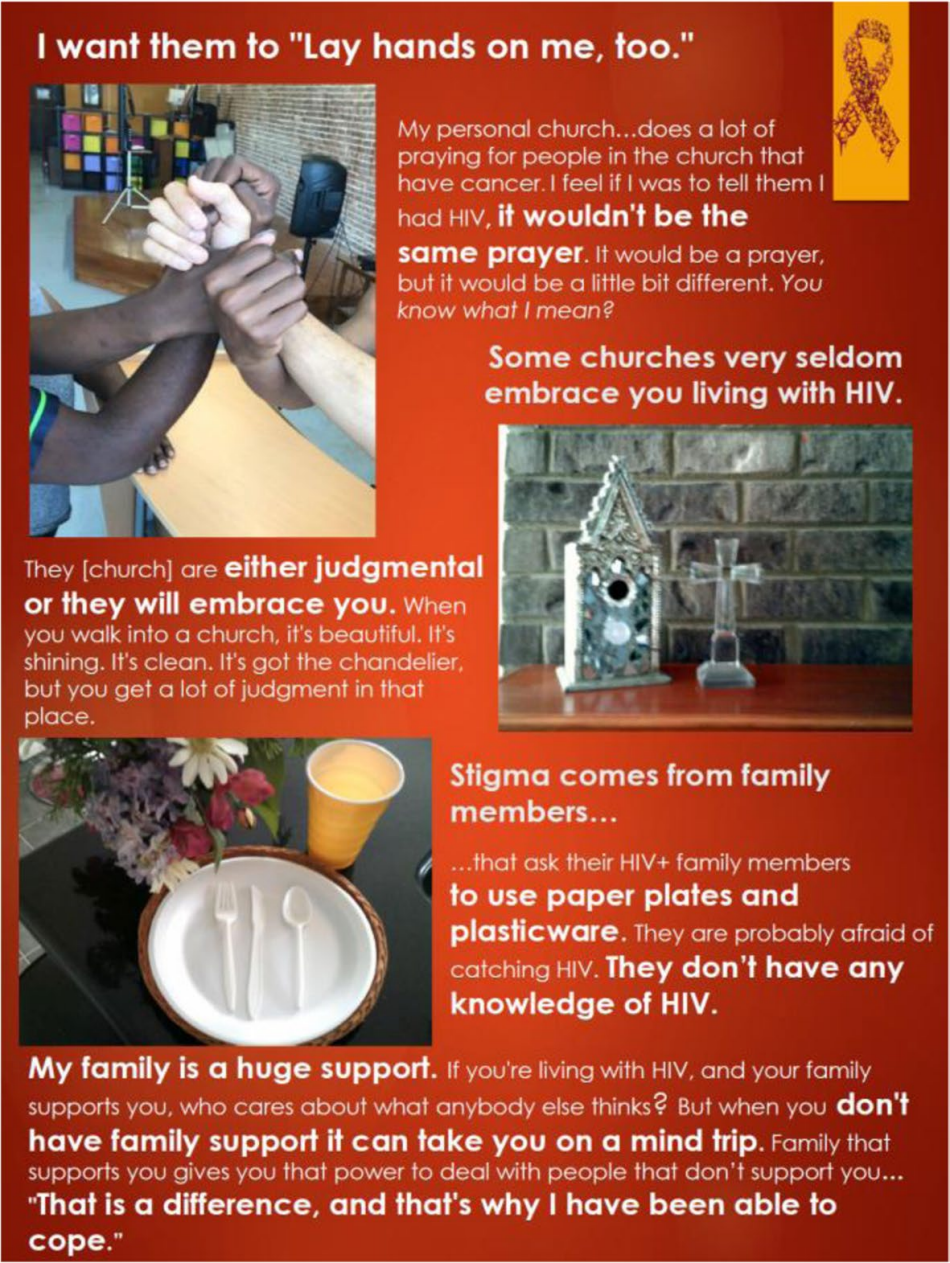
I want them to lay hands on me, too

Several participants identified the Black church as a source of support for themselves currently or their family historically. For example, participant #12 talked about his commitment to his church and how he wanted to provide education to his pastor and to the congregation about HIV. He has made educating his faith-based institution and other faith-based institutions his life’s mantra. He strongly believes in using his own personal face and story to help reduce negative perceptions about people living with HIV. Others wanted to see their churches as supportive but saw limitations to this. For example, participant #8 said their church prayed for people with cancer and although they hoped that HIV would receive the same level of care – they admitted, “it wouldn’t be the same prayer.” (Fig 3). Participant #9 described how their church had a sign indicating “love without limits” but that it was “a lie” because that church would not allow PLWH to come and speak there.

Others talked directly about discrimination at the church. For example, participant #16 said, “When I was first diagnosed, I went to my Pastor. He pretty much turned his back on me after I served on the usher board for four years. I felt like I could go to him.” Participant #28 (transwoman) added that they were called names (“faggot, punk, boy, sissy”) in the church. Others, like participant #45, described how he expected protection but instead experienced abuse by church members.

When I was small, all we did was stay in the church, from sunup to sundown … that’s where I was supposed to have been protected. I was not protected, I was abused. Just evil in my eyesight because the things I went through … It still bothers me right now, so that’s where a little of my stigma comes from, the church, because there’s a lot of things that happened to me in church.

The Black Church was not a single experience for people, however. Some received support, others wanted it, but didn’t find it, and others, were hurt by the church – and regardless of experience, many still wanted and held hope that the church could be “open and understanding of people’s lives” (Participant #28). Participant #8 described the complexities and ironies best when he said:

They [church] are either judgmental or they will embrace you. When you walk into a church, it’s beautiful. It’s shining. It’s clean. It’s got the chandelier, you get a lot of judgment in that place.

### Self as inner support and stigma

Self-strength or resilience was a source of support for participants, but internalized stigma also manifested among participants – and challenged their ability to protect themselves from stigma. Several participants said they believed that they did something wrong to get HIV and thus, hid it from people. For instance, participant #15 said “I was mad at myself because I allowed myself to be in this situation, so I was blaming me for having HIV. I waited about 13 years before I told anyone.” Participant #23 noted he could internalize stigma by saying it could get under his skin and then he would “start crumbling.” Similarly, participant #44 showed a picture of a trash can to show that stigma could make one feel “unimportant” and “like trash.”

**Fig. 4:**
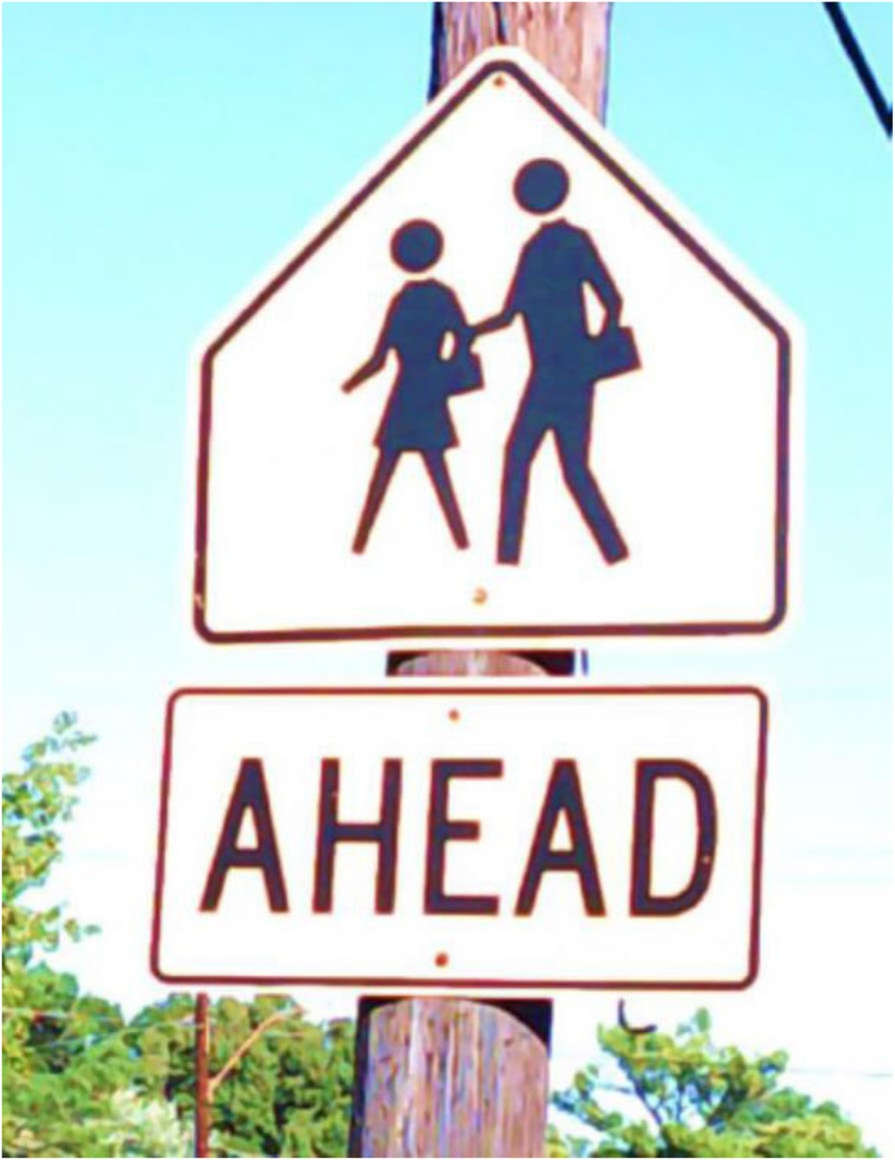
Be proud of yourself and move ahead, because it’s a future before it’s ever going to be a past again

Other participants relied deeply on their internal resources to overcome stigma. This was a particular key theme among transgender participants as compared to cisgender participants. For example, this theme was apparent among discussions of ten participants who identified as transgender in the study and twenty-three who identified as cisgender so it was a notable difference. These participants also commonly noted the layered and intersectional stigma regarding their HIV and gender status, like participant #24:

We all experience some type of trauma, whether it’s emotional, mental, or physical trauma. Having HIV or having any STD, you being trans or gay, it’s not just something that’s going to stop. It’s always going to be a discrimination behind it or somebody criticizing what you do. I took a picture of the sign to remind us that you always can move ahead (Fig. 4).

Participant #27 discussed police violence – and specifically police “dumping” trans people across the state border, but noted, “we’re here to stay, and you cannot take us down.”

Others also noted moving forward amid challenges via inner strength and positivity. Participant #22 noted that people may judge or “put stigma on me” but that “No matter what, we all are human. We’re all valid, we all have a voice in this world, and we all have special talents … [Stigma] is not me. I’m me. I’m an individual and I stand alone.” Participant #22 also said it was important to “demand respect,” an idea shared by others. Participant #49 said he was “strong, a fighter” and Participant #31 defined herself as a “hero” explaining:

I love myself and I love waking up seeing myself as a girl. I want them to know that’s a hero. A black African hero. Being trans, they can know that you can survive for you, you can still live, because you got some trans that don’t care about life.

Similarly, participant #43 said that taking the shame off of HIV helped him and others to gain “knowledge, a sustainable life, and a good life.”

## Discussion

The Snap Out Stigma (SOS) photovoice project created a safe space for PLWH to share lived experiences of HIV stigma via photography in group discussions and individual interviews. Discussions and photo displays revealed the intersection of stigma and support with four emerging themes: medical, social support, church, and self. In general, participants discussed disclosing HIV status to loved ones, revealing child sexual trauma, being outed for sexuality, and being rejected from religious homes. In contrast, there were displays of personal growth and self-acceptance living with HIV.

Our findings of medical care providing sources of support as well as stigmatization is consistent with other studies reporting medication adherence among PLWH [38]. Participants in the current project spoke of antiretroviral therapy as a direct lifeline and how pill-taking is essential as a lifesaving support tool. Similarly, another U.S. sample of PLWH endorsed how treatment empowered them to take control of their health [29]. Other studies reinforced a positive relationship with quality medical treatment and health outcomes. However, several participants in SOS lamented on the fear and worry of being recognized by healthcare personnel in clinic spaces which served as a barrier to adhering to routine medical appointments. Being ‘outted’ has been identified in other work as a deterrent to receipt of medical support and prevention services [39].

Social support has been well-documented in the literature as a mechanism of support for PLWH, coping with HIV disease, and medication adherence. Our participants discussed how close friends and family facilitated disclosing their HIV status and encouraged them to take their medications to live a heathier fulfilling life. Other research among Memphis Ryan White Program clients found that 94% of adults prescribed antiretroviral therapy reported disclosing their HIV status to someone [40]. However, 20% of those people that were prescribed antiretroviral therapy reported lack of support from their church.

Several of the participants in this study expressed the importance of U.S. southern churches in their current daily lives and upbringing in shaping their beliefs and moral character. Participants discussed how the church can be an instrumental vehicle for providing HIV education to congregations and surrounding community to minimize stigma. Other studies among Black MSM echo a desire to attend worship services and participate in other activities without judgment [41]. Young Black MSM recommended reducing homosexuality stigma by increasing interpersonal and institutional acceptance [41]. Similar to PLWH in SOS, Black MSM in Powell et al. reported feeling uncomfortable and isolated which serve as barriers to regular church attendance. Participants in our study expressed hope of potential for growth through church-centered education and learning.

Finally, self as a site of inner strength and support in the current study is congruent with other literature. A technologically delivered stigma reduction intervention conducted in the Deep South improved self-esteem and coping self-efficacy and decreased internalized HIV-related stigma among PLWH [42]. There is a growing body of literature supporting empowerment and resilience among PLWH which dovetails several of the participants articulating their strength and growth with acceptance of HIV. However, at different points along the continuum of acceptance our participants revealed self-blame and feeling unworthy of love. Other research conducted among gay men in the U.S. South demonstrates that when stigma is internalized by PLWH it leads to poorer health outcomes, engagement in risk behaviors, contemplation of suicide, and avoidance of intimate relationships [43].

This study is not without limitations. First, internalizations of HIV stigma were not as pronounced as we originally had expected among PLWH knowing the prevalence of stigma in the U.S. southern region. This finding could be attributed to many of the participants knowing their HIV status for several years and most were not new diagnoses which allowed some time for greater acceptance and processing. Also, our inclusive recruitment approach and less rigid eligibility criteria resulted in a wider age range. Our findings may not be consistent with newly diagnosed adolescent and young adults who are driving HIV incidence in Memphis, TN. Although we found participants to have a more positive outlook about their individual diagnoses, efforts are still needed to support disclosure and minimize emotional distress.

## Conclusions

Photovoice facilitated numerous dialogue topics ranging from healing and recovery, managing HIV disease, to overcoming underlying factors of social determinants of HIV. The findings highlight the complexity of stigma in people’s lives and how future work needs to both foster growth in these areas and continue to push for structural change as high burden communities work toward Ending the HIV Epidemic. We identified trauma-informed growth as a priority area of future programs for PLWH. The narratives of self-protection and self-preservation may be focusing on coping mechanisms to continue living a full life with HIV.

## Supplementary Information

**Additional file 1.** Group Discussion and Individual Interview Guide.

## Data Availability

The qualitative data analyzed during the current project are not publicly available due to our lack of non-data sharing agreement during the acquisition of data but are available from the corresponding author on reasonable request.

## Abbreviations

C2P: Connect to Protect
EHE: Ending the HIV Epidemic
H-CAP: HIV Care and Prevention Group
HIV: Human Immunodeficiency Virus
LGBTQ+: Lesbian, gay, bisexual, transgender and queer or questioning
PLWH: People living with HIV
MSA: Metropolitan statistical area
MSM: Men who have sex with men
SOS: Snap Out Stigma
STD: Sexually transmitted disease
WLWH: Women living with HIV
