# Supplementary material for: Snap out stigma photovoice project in the U.S. South
Source: BMC Health Serv Res. 2022 Jun 20;22:795. doi: 10.1186/s12913-022-08147-3 (PMC9208191; doi:10.1186/s12913-022-08147-3)
Supplement: Supplementary file 1 — Additional file 1. Group Discussion and Individual Interview Guide. [file 12913_2022_8147_MOESM1_ESM.docx]

Group Discussion Guide

Preface: These questions are in regards to the photos taken.

1. What do you **s**ee here?
2. What is **h**appening?
3. How does this relate to **o**ur lives?
4. **W**hy does the problem or strength **e**xist?
5. What can we **d**o about it?

One-on-One Interview Guide

1. Now that you have a basic understanding of internalized stigma, explain to me how this has affected you personally?
2. What is the role of church in your life?
3. What role has the churched play in internalizing HIV-related stigma?
4. How would you describe your experience participating in the Project SNAP to someone else?
5. What did you learn about the role of the church in your life and how you internalize stigma?
6. What could the church do to be more supportive in reducing internalized stigma?
7. Describe the experience of watching other people view your photos.
